# Expression of YAP/TAZ in Keratocystic Odontogenic Tumors and Its Possible Association with Proliferative Behavior

**DOI:** 10.1155/2017/4624890

**Published:** 2017-04-23

**Authors:** Qi-Wen Man, Yan-Qi Ma, Jin-Yuan Liu, Yi Zhao, Bing Liu, Yi-Fang Zhao

**Affiliations:** ^1^The State Key Laboratory Breeding Base of Basic Science of Stomatology and Key Laboratory of Oral Biomedicine Ministry of Education, School and Hospital of Stomatology, Wuhan University, Wuhan, China; ^2^Department of Prosthodontics, School and Hospital of Stomatology, Wuhan University, Wuhan, China; ^3^Department of Oral and Maxillofacial Surgery, School and Hospital of Stomatology, Wuhan University, Wuhan, China

## Abstract

The aim of this study is to clarify whether YAP/TAZ is involved in the pathogenesis and proliferative growth of keratocystic odontogenic tumor (KCOT). The expression levels of YAP/TAZ and downstream proteins and genes in normal oral mucosa (OM) and KCOT were determined and compared by immunohistochemistry and real-time quantitative PCR. The results showed that the expression of YAP/TAZ and downstream proteins (Cyr61, CTGF) was significantly upregulated in KCOT with upregulation of Ki-67 compared to OM. Importantly, the mRNA levels of transcription factors (TEAD1, TEAD4, and RUNX2) and cell cycle related genes (CDK2, PCNA), which interact with the transcriptional coactivators YAP/TAZ, are also upregulated in the KCOT. In addition, the results from Spearman rank correlation test revealed the close relationship between YAP/TAZ and Ki-67, which was further evidenced by double-labelling immunofluorescence that revealed a synchronous distribution for YAP/TAZ with Ki-67 in KCOT samples. All the data suggested YAP/TAZ might be involved in the proliferative behavior of KCOT.

## 1. Introduction

Keratocystic odontogenic tumor (KCOT) is formerly known as odontogenic keratocyst (OKC) [1]. In 2005, OKC was reclassified as a benign neoplasm of odontogenic origin under the name of “keratocystic odontogenic tumor” by the World Health Organization [2]. In the cysts and tumors of jaw bones, KCOT has been reported as the second most common in the Chinese population [3]. The KCOT has some particular histologic features compared with other odontogenic lesions: parakeratinized stratified cell layers, daughter cysts, budding proliferation of the epithelium, and the formation of the islands of odontogenic epithelium [4]. Quite unexpectedly, the KCOT has a great potential to recur and the recurrence rate ranged from 0 to 100 percent due to different observation periods and treatments [5]. It has been shown that the expressions of cellular proliferative markers (Ki-67, PCNA, and Cyclin D1) of the epithelium were consistent with the aggressive features of the KCOT [6, 7].

Recently, the aberrant activation of the transcriptional regulators YAP/TAZ was proved to contribute to the initiation and progression of a range of tumors [8]. YAP/TAZ transcriptional activity is dependent on their recruitment to the nucleus, which promotes binding to a range of transcription factors, most notably the TEAD family [9]. The transcription complexes regulate expression of a number of downstream target genes such as CTGF and Cyr61 [8, 9]. YAP/TAZ-directed transcription promotes cell proliferation, prosurvival, and cell migration signals, all of which contribute to the protumorigenic roles of YAP/TAZ [8]. Multiple signaling events restrict YAP/TAZ from the nucleus, the best characterized of which are signals mediated by the Hippo pathway [9]. Besides, the mechanical cues and signals that affect cytoskeletal dynamics, such as those signals transduced by G-protein coupled receptors, also control YAP/TAZ localization [10]. In the oral and maxillofacial regions, dysregulated YAP/TAZ activity has been reported to be implicated in head and neck tumors [11]. However, the expression of YAP/TAZ and its downstream proteins in KCOT is still not clear.

In the present study, therefore, we detected the expression of YAP/TAZ, Cyr61, CTGF, Ki-67, YAP, and TAZ in clinical samples of KCOT and OM using immunohistochemical, immunofluorescence staining, and western blot analysis, respectively. The transcription factors (TEAD1, TEAD4, and RUNX2), cell cycle related genes (CDK2, PCNA), YAP, and TAZ genes were compared using RT-qPCR analysis. The correlation between the nucleus expression level YAP/TAZ and the cell proliferative marker (Ki-67) in KCOT was also explored using Spearman rank correlation and double immunofluorescence staining. This is the first study that has evaluated the dysregulated status of YAP/TAZ transcriptional activity in KCOT and explored its possible association with the growth potential.

## 2. Materials and Methods

### 2.1. Specimens

Twenty-one samples of human KCOT and eight samples of human oral mucosa (OM) were collected after operations at the Hospital of Stomatology, Wuhan University. The procedures were implemented according to the National Institutes of Health guidelines regarding the use of human tissues. The study was approved by the review board of the Ethics Committee of Hospital of Stomatology, Wuhan University. The pathological diagnosis of KCOT was confirmed by haematoxylin and eosin staining based on the criteria described by the WHO classification. All samples were fixed immediately in 4% buffered paraformaldehyde and routinely embedded in paraffin; then sections approximately 4 *μ*m thick were cut from the paraffin blocks for immunostaining.

### 2.2. Immunohistochemistry

Immunostaining was carried out with the streptavidin biotin method on dewaxed tissue sections as previously described [12]. Briefly, 4 *μ*m thick sections were processed for subsequent histological and immunohistochemical study. Primary antibodies were YAP/TAZ (Cell Signaling Technology, rabbit, 1 : 50, #8418), Cyr61 (Proteintech, rabbit, 1 : 200, #26689-1-AP), CTGF (Proteintech, rabbit, 1 : 200, #23936-1-AP), Ki-67 (Abcam, mouse, 1 : 200, #ab8191), YAP (Cell Signaling Technology, rabbit, 1 : 100, #14074), and TAZ (Cell Signaling Technology, rabbit, 1 : 100, #4883). To visualize the reaction, 3,30-diaminobenzidine (DAB Chromogen, DakoCytomation) was used. Finally, slides were counterstained by Mayer's hematoxylin for 10 seconds. A brown colored cytoplasmic reaction was considered to indicate immunopositivity for Cyr61 and CTGF. Overlapping nuclei sections were considered positive immunopositivity for YAP/TAZ, Ki-67, YAP, and TAZ. For quantization, the numbers of stained cells were counted at an original magnification 200x in 5 random fields by 2 investigators in the cyst wall layer regions. For YAP/TAZ, Ki-67, YAP, and TAZ quantization, the immunoscore was expressed as the percentage of stained cells. For Cyr61 and CTGF evaluation, the staining scores were evaluated as the sums of staining intensity (0 = no staining; 1 = mild staining; 2 = moderate staining; and 3 = intense staining) and the percentage of stained cells (0 ≤ 10%; 1 = 11–25%; 2 = 26–50%; 3 = 51–75%; and 4 > 75% of the KCOT and OM epithelial cells).

### 2.3. Western Blotting

Proteins were extracted from RNA later-preserved tissues from KCOT and OM. The tissue homogenate was prepared to disrupt the cell membranes under precooling conditions. After centrifugation at the speed of 14,000*g* for 10 minutes, the protein containing supernatant was collected and the concentration of the protein was estimated using the BCA assay (Pierce, Rockford, IL, USA). Supernatants of the KCOT and OM collections were boiled for 5 minutes in SDS gel loading buffer before electrophoresis. 20 *μ*g of protein was separated on 10% SDS-polyacrylamide gels and transferred onto polyvinylidene fluoride (PVDF) membranes (Roche Applied Science). The blots were blocked overnight with 5% nonfat dry milk and incubated with primary antibody at dilutions YAP (Cell Signaling Technology, rabbit, 1 : 1000, #14074), TAZ (Cell Signaling Technology, rabbit, 1 : 2000, #4883), and GAPDH (Santa Cruz Biotechnology, mouse, 1 : 1000, #sc-32233). Immunoblots were detected by HRP-conjugated secondary antibodies (Pierce) using the chemiluminescence kit (Pierce). The proteins were detected by the ECL detection system and the light emitted was captured on film.

### 2.4. Real-Time Quantitative Polymerase Chain Reaction (RT-qPCR)

Total RNA was isolated from tissues of four cases of KCOT and three cases of OM by using an RNeasy Mini kit (Qiagen, Hilden, Germany) according to the manufacturer's instructions. The RNA (2 *μ*g) was transcribed into cDNA using the PrimeScript 1st Strand cDNA Synthesis Kit (Takara, Otsu, Japan). The transcribed product was amplified by polymerase chain reaction (PCR) into a final volume of 20 *μ*L by using the following sense and antisense primers: TEAD1: 5′-ATGGAAAGGATGAGTGACTCTGC-3′ and 5′-TCCCACATGGTGGATAGATAGC-3′; TEAD4: 5′-GAACGGGGACCCTCCAATG-3′ and 5′-GCGAGCATACTCTGTCTCAAC-3′; RUNX2: 5′-TGGTTACTGTCATGGCGGGTA-3′ and 5′-TCTCAGATCGTTGAACCTTGCTA-3′; CDK2: 5′-CCAGGAGTTACTTCTATGCCTGA-3′ and 5′-TTCATCCAGGGGAGGTACAAC-3′; PCNA: 5′-CCTGCTGGGATATTAGCTCCA-3′ and 5′-CAGCGGTAGGTGTCGAAGC-3′; YAP: 5′-ACGTTCATCTGGGACAGCAT-3′ and 5′-GTTGGGAGATGGCAAAGACA-3′; TAZ: 5′-ATTCATCGCCTTCCTAGGGT-3′ and reverse 5′-GGCTGGGAGATGACCTTCAC-3′; GAPDH: 5′-CCATGTTCGTCATGGGTGTGAACCA-3′ and 5′-GCCAGTAGAGGCAGGGATGATGTTC-3′. GAPDH was selected as the internal control for each experiment.

### 2.5. Double-Labelling Immunofluorescence

Double-labelling immunofluorescence was done as described previously [12]. Briefly, 4 *μ*m thick sections were processed for subsequent immunofluorescence staining. The primary antibodies for double-labelling immunofluorescence were YAP/TAZ (Cell Signaling Technology, rabbit, 1 : 50, #8418), Cyr61 (Proteintech, rabbit, 1 : 100, #26689-1-AP), CTGF (Proteintech, rabbit, 1 : 100, #23936-1-AP), and Ki-67 (Abcam, mouse, 1 : 100, #ab8191). Double-labelling immunofluorescence for YAP/TAZ and DAPI, YAP/TAZ and Ki-67, and Cyr61 and CTGF was performed in samples of both KCOT and OM. The nuclei were counterstained with 40,6-diamidino-2-phenylindole (DAPI) for 10 minutes at room temperature. The slides were stored in −20°C for later detection.

## 3. Results

### 3.1. Upregulated Expression of YAP/TAZ, Cyr61, CTGF, Ki-67, YAP, and TAZ Proteins in KCOT

**Figure 1:**
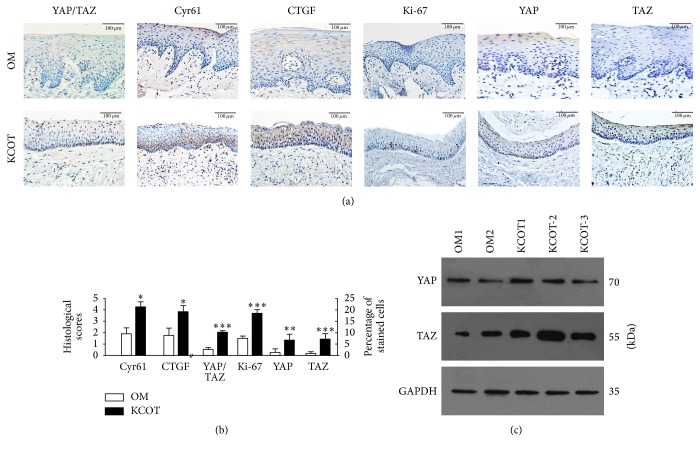
(a) YAP/TAZ, Cyr61, CTGF, Ki-67, YAP, and TAZ expression in normal oral mucosa and keratocystic odontogenic tumors. (b) Immunostaining scores for indicated markers in samples from both normal oral mucosa and keratocystic odontogenic tumors. All values are mean (SEM), ^*∗*^*p* < 0.05, ^*∗∗*^*p* < 0.01, and ^*∗∗∗*^*p* < 0.001. (c) Western blotting for YAP, TAZ, and GAPDH in the samples from oral mucosa and keratocystic odontogenic tumor.

To investigate the activation status of YAP/TAZ in KCOT, the expression of the YAP/TAZ, and the downstream proteins Cyr61, CTGF was explored and compared in the twenty-one KCOT samples and eight normal oral mucosa samples by immunochemistry. In the sections of KCOT, the tested proteins including YAP/TAZ, CTGF, Cyr61, Ki-67, YAP, and TAZ were expressed in the epithelial layers (Figure 1(a)). Specifically, the YAP/TAZ and Ki-67 were mainly located in the nucleus of the basal cell layers of KCOT; YAP and TAZ were located in the nucleus of the basal and superficial epithelial layers of KCOT. Additionally, TAZ proteins were also frequently expressed within the stromal component of KCOTs. Also, the immunofluorescence staining of the YAP/TAZ in KCOT showed overlapping with DAPI indicating the nucleus expression. However, few overlaps of YAP/TAZ and DAPI were observed in the OM (see Supplementary Figure  1 in Supplementary Material available online at https://doi.org/10.1155/2017/4624890, white arrow). The Cyr61 and CTGF were mainly localized in the cytoplasm of the epithelium of KCOT. In contrast, the immunoreactivities of the YAP/TAZ and downstream proteins (Cyr61, CTGF) in KCOT showed more expression compared with these from OM. More importantly, the data further confirmed that the expressions of YAP/TAZ (*p* < 0.0001), Cyr61 (*p* = 0.0103), CTGF (*p* = 0.0467), Ki-67 (*p* = 0.0006), YAP (*p* = 0.0073), and TAZ (*p* < 0.0001) were significantly greater in samples of KCOT than those from OM (Figure 1(b)).

### 3.2. Western Blot Analysis

To confirm the presence of YAP and TAZ proteins in the KCOT and OM, western blotting of YAP and TAZ was performed using the tissue lysates from two cases of OM and three cases of KCOTs. The results showed the bands of the expected sizes of 70 kDa (YAP) and 55 kDa (TAZ) for all the analysed samples. The band for 55 kDa (TAZ) protein in OM was weaker than that in KCOT samples (Figure 1(c)).

### 3.3. Upregulated mRNA Expression of Transcription Factors (TEAD1, TEAD4, and RUNX2), Cell Cycle Related Genes (CDK2 and PCNA), YAP, and TAZ in KCOT

**Figure 2:**
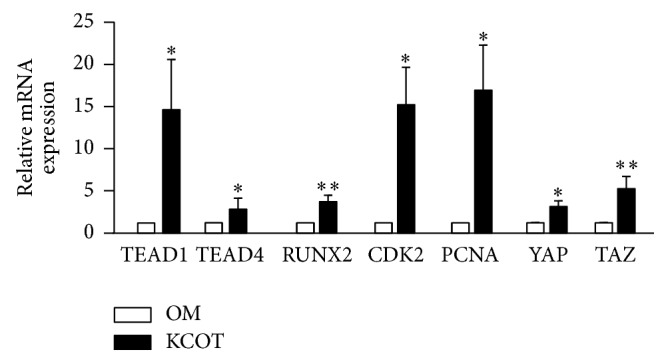
The mRNA expression levels of TEAD1, TEAD4, RUNX2, CDK2, PCNA, YAP, and TAZ in samples from both keratocystic odontogenic tumors (solid bars) and oral mucosa (open bars) were evaluated by real-time qPCR analysis. Data are mean (SEM). ^*∗*^*p* < 0.05 and ^*∗∗*^*p* < 0.01 compared with radicular cysts.

To investigate the expression levels of transcription factors and cell cycle related genes, the mRNA levels of TEAD1, TEAD4, RUNX2, CDK2, PCNA, YAP, and TAZ were explored in the samples of KCOT (*n* = 4) and normal mucosa (*n* = 3) by real-time qPCR analysis. The results showed that the tested genes were expressed in KCOT samples. The mRNA expressions of the TEAD1 (*p* = 0.0196), TEAD4 (*p* = 0.0351), RUNX2 (*p* = 0.0015), CDK2 (*p* = 0.0209), PCNA (*p* = 0.0263), YAP (*p* = 0.0461), and TAZ (*p* = 0.0011) were significantly higher in samples from KCOT than those from OM (Figure 2).

### 3.4. Synchronous Distribution for YAP/TAZ and Proliferative Marker (Ki-67) in KCOT

**Figure 3:**
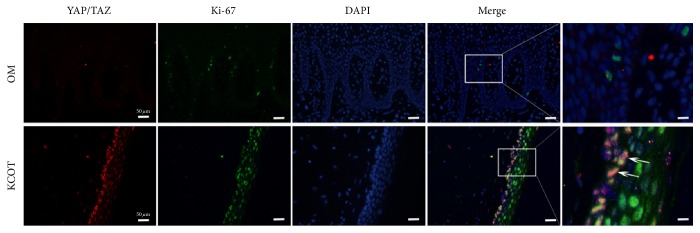
Double-labelling immunofluorescence staining for YAP/TAZ (red) and Ki-67 (green) in specimens of keratocystic odontogenic tumors and oral mucosa. The white arrow indicated the colocalisations of YAP/TAZ with Ki-67 signals in keratocystic odontogenic tumors.

To validate the correlation between YAP/TAZ activation and growth potential further, double-labelling immunofluorescence analysis for YAP/TAZ and Ki-67 was made on samples from KCOT and OM. As is shown in Figure 3, the characteristic YAP/TAZ dots were colocalised in the nucleus of the KCOT epithelium. The results showed that the YAP/TAZ was transported into the nucleus. There were also several colocalisations of YAP/TAZ with the Ki-67 signals in the sections of KCOT (Figure 3, white arrow). However, immunofluorescent signals for YAP/TAZ or Ki-67 were rarely expressed in the epithelium of samples from oral mucosa.

### 3.5. Synchronous Distribution for Cyr61 and CTGF in KCOT and OM

**Figure 4:**
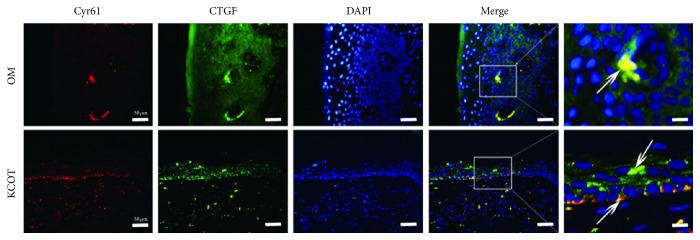
Double-labelling immunofluorescence staining for Cyr61 (red) and CTGF (green) in samples of keratocystic odontogenic tumors and oral mucosa. The white arrow indicated the colocalisations of Cyr61 and CTGF signals both in keratocystic odontogenic tumors and in oral mucosa.

To validate the correlation between downstream molecules of YAP/TAZ, double-labelling immunofluorescence analysis for Cyr61 and CTGF was made on samples from KCOT and OM. As is shown in Figure 4, the Cyr61 and CTGF signals are both present in the basal layers of KCOT and OM. Several overlapping expressions of Cyr61 and CTGF were detected in both samples of KCOT and OM (Figure 4, white arrow).

### 3.6. Correlation between Staining of YAP/TAZ, CTGF, Cyr61, and Ki-67 in KCOT

To further work out the potential significance of the activation of YAP/TAZ related to the proliferative characteristics of KCOT, the relationship was then explored between the histoscores of YAP/TAZ, Cyr61, CTGF, and proliferative marker (Ki-67) in KCOT. The results showed significant correlation of the immunostaining of YAP/TAZ and Ki-67 (Table 1, *p* = 0.0412). Besides, the immunostaining of YAP/TAZ downstream-related proteins (Cyr61, CTGF) was also shown to correlate with Ki-67 staining (Table 1, *p* = 0.0291, *p* = 0.0003). All these findings suggested a possible link between activation of YAP/TAZ and growth potential in KCOT.

## 4. Discussion

First studied in* Drosophila*, YAP/TAZ was proved to play an important role in the control of organ size during development [13]. In several adult organs, YAP/TAZ not only play important roles for normal homeostasis but also are critical to promoting tissue repair upon injury [14–16]. Recently, accumulating evidences have supported that YAP and TAZ are oncogenes in various mammalian cells [17]. Genome-wide analysis using mouse model of hepatocellular carcinomas showed chromosome amplification at 9qA1, which was syntenic to chromosome region 11q22 in human. YAP gene was in the amplified region and individually oncogenic [18]. YAP gene was also amplified in human cancers such as oral squamous cell carcinomas and medulloblastomas [19, 20]. TAZ, also called WWTR1 (WW-domain containing transcriptional regulator 1), was first identified as a 14-3-3 binding protein in the year 2000 [21]. The human TAZ gene, located at 3q23-q24, encoded a 43 kDa protein. TAZ was expressed in various tissues, but not thymus and peripheral blood leukocytes, and TAZ was also amplified in various human cancers such as breast cancers, non-small cell lung cancers, and gastric cancers [21].

Oral soft tissues were complicated biologic systems with the components responding to various physiologic stresses. These stresses included compression, hydrodynamic forces, elongation, friction, and shear stress generated during mastication, saliva flow, pronunciation, tooth-brushing, facial expression, and tooth eruption [22]. In the disease state such as jaw cysts, the cyst wall was exposed to hydrostatic pressures, osmotic pressures, and occlusal forces [23]. At cellular levels, cells could perceive external mechanical signals through various “mechanical sensors.” These “mechanical sensors” include integrin based cell-matrix adhesions, stretch-modulated ion channels, and cell-cell junctions [24]. After sensing the mechanical cues, actin cytoskeleton could be activated, which is regulated by the RHO family of GTPases. Subsequently, contractile filamentous actin structures (F-actin) sustain YAP and TAZ nuclear localization and activity [24]. YAP and TAZ had been shown to play important roles in organ development of oral region. For instance, *α*E-catenin was an integral component of adherens junctions, where it linked cadherins to the actin cytoskeleton [25]. In the oral cavity, an elegant study has shown that *α*E-catenin restricts YAP/TAZ activity to establish a low proliferative cell group called enamel knot, which is a putative signaling center regulating tooth development [25]. Using YAP constitutive activation mice, other researchers also found that overexpression of YAP resulted in deformed tooth morphogenesis with widened dental lamina and mislocated enamel knot [26]. However, the expression status of YAP/TAZ in KCOT, a benign odontogenic tumor which arose from dental lamina and featured by mechanical loading, is still unclear [1, 4, 23].

YAP/TAZ dysregulation is wide spread in various human tumors, where YAP/TAZ have been shown to be indispensable for tumor initiation, progression, or metastasis [27–31]. YAP/TAZ are transcriptional coactivators that shuttle between the cytoplasm and the nucleus, where they interact with other transcription factors, especially TEA domain family members (TEAD) [28]. YAP/TAZ/TEAD control the expression of their targets genes such as Cyr61 and CTGF [28]. Sustained nucleus activation of YAP/TAZ promotes aberrant cell proliferation and overcome anoikis [32]. In this study, the immunoreactivities of YAP/TAZ, Cyr61, CTGF, Ki-67, YAP, and TAZ were significantly upregulated in KCOT. Besides, the mRNA level of the transcription factors such as TEAD1, TEAD4, RUNX2, YAP, and TAZ was also significantly upregulated in KCOT. All these findings suggest possible nucleus activation of YAP/TAZ in KCOT.

It has been widely known that KCOT owns a distinct growth behavior compared with the other kinds of jaw cysts and normal tissue [1]. The lining epithelium is proved to have a higher proliferative potential [33]. An emerging evidence is that aberrant expression of YAP/TAZ was possibly associated with the upregulated expression of tumor cell's proliferative markers [28]. In line with previous studies, our results also showed that the expression of Ki-67 was significantly higher in the epithelial layers than those in normal oral mucosa [34]. The significant relationship between the YAP/TAZ, Cyr61, CTGF, and Ki-67 was verified by Spearman rank correlation. The results from the double-labelling immunofluorescence also showed the partially synchronous distribution for YAP/TAZ and proliferative marker (Ki-67) in KCOT, which indicated the possible association between the YAP/TAZ activation and proliferative activity in KCOT.

## 5. Conclusions

In summary of the present study, the activation status of YAP/TAZ was investigated in the KCOT and also its possible association with aberrant proliferation characteristics of the KCOT epithelium was studied. As the activation status of YAP/TAZ may favor the unusual growth potential of KCOT, therefore, we have partially revealed the proliferative nature and the underlying mechanisms of KCOT. However, further investigation is still needed to uncover the precise molecular events behind the YAP/TAZ activation in KCOT.

## Supplementary Material

To further detect the YAP/TAZ protein expression locations in keratocystic odontogenic tumors. Double-labelling immunofluorescence staining for YAP/TAZ (red) and DAPI in specimens of keratocystic odontogenic tumors and oral mucosa was carried out. The white arrows indicated the colocalisations of YAP/TAZ with DAPI signals in keratocystic odontogenic tumors.

## Figures and Tables

**Table 1:** Spearman rank analysis of the immunoreactivities. The data are given as  *p*  values.

Marker	YAP/TAZ	Cyr61	CTGF	Ki-67
YAP/TAZ	—	0.0140	0.0250	0.0412
Cyr61	—	—	0.1515	0.0291
CTGF	—	—	—	0.0003
