# Supplementary material for: Expression of YAP/TAZ in Keratocystic Odontogenic Tumors and Its Possible Association with Proliferative Behavior
Source: Biomed Res Int. 2017 Apr 23;2017:4624890. doi: 10.1155/2017/4624890 (PMC5420425; doi:10.1155/2017/4624890)
Supplement: Supplementary file 1 — To further detect the YAP/TAZ protein expression locations in keratocystic odontogenic tumors. Double-labelling immunofluorescence staining for YAP/TAZ (red) and DAPI in specimens of keratocystic odontogenic tumors and oral mucosa was carried out. The white arrows indicated the colocalisations of YAP/TAZ with DAPI signals in keratocystic odontogenic tumors. [file 4624890.f1.doc]

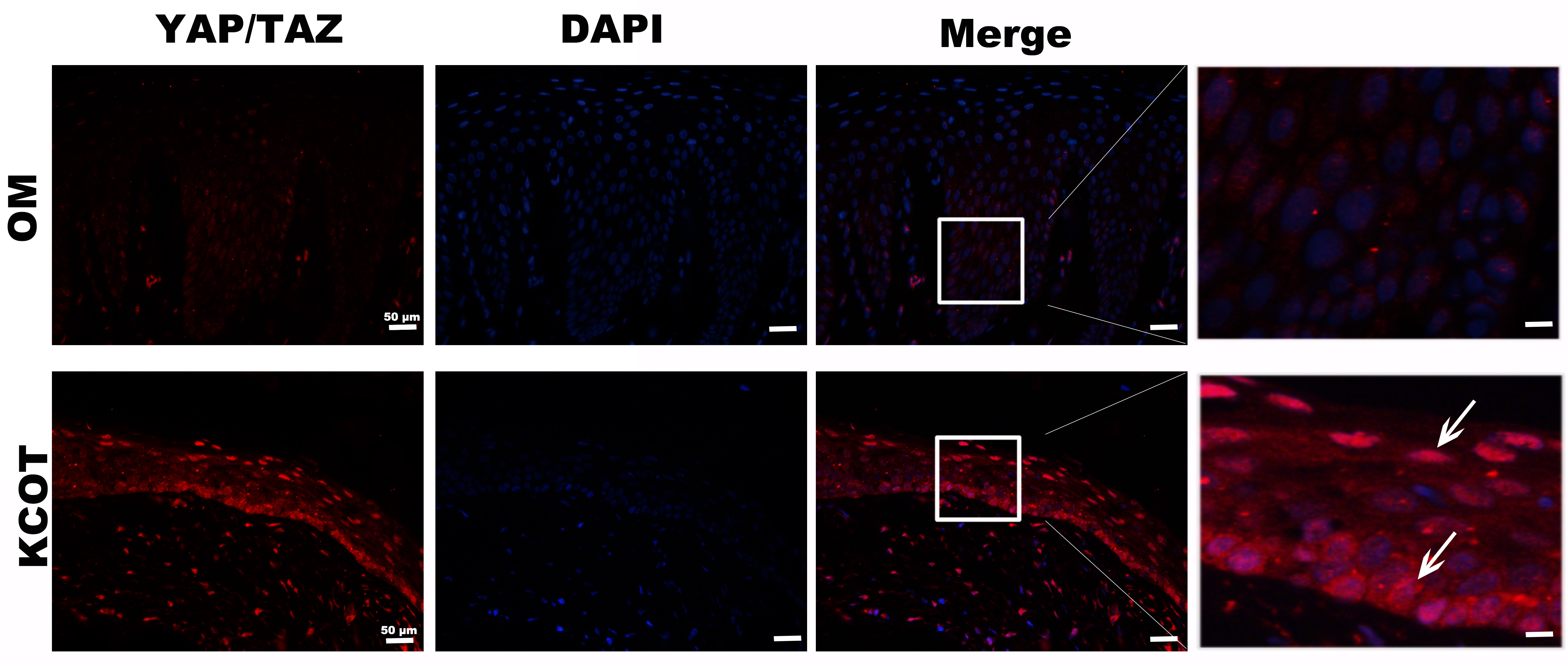


Supplementary Figure legend

To further detect the YAP/TAZ protein expression locations in keratocystic odontogenic tumors. Double-labelling immunofluorescence staining for YAP/TAZ (red) and DAPI in specimens of keratocystic odontogenic tumors and oral mucosa was carried out. The white arrows indicated the colocalisations of YAP/TAZ with DAPI signals in keratocystic odontogenic tumors.
